# Mixed Effects of Elevated *p*CO_2_ on Fertilisation, Larval and Juvenile Development and Adult Responses in the Mobile Subtidal Scallop *Mimachlamys asperrima* (Lamarck, 1819)

**DOI:** 10.1371/journal.pone.0093649

**Published:** 2014-04-14

**Authors:** Elliot Scanes, Laura M. Parker, Wayne A. O’Connor, Pauline M. Ross

**Affiliations:** 1 School of Science and Health, University of Western Sydney, Sydney, New South Wales, Australia; 2 Industry and Investment NSW, Port Stephens Fisheries Institute, Taylors Beach, New South Wales, Australia; University of Hong Kong, Hong Kong

## Abstract

Ocean acidification is predicted to have severe consequences for calcifying marine organisms especially molluscs. Recent studies, however, have found that molluscs in marine environments with naturally elevated or fluctuating CO_2_ or with an active, high metabolic rate lifestyle may have a capacity to acclimate and be resilient to exposures of elevated environmental *p*CO_2_. The aim of this study was to determine the effects of near future concentrations of elevated *p*CO_2_ on the larval and adult stages of the mobile doughboy scallop, *Mimachlamys asperrima* from a subtidal and stable physio-chemical environment. It was found that fertilisation and the shell length of early larval stages of *M. asperrima* decreased as *p*CO_2_ increased, however, there were less pronounced effects of elevated *p*CO_2_ on the shell length of later larval stages, with high *p*CO_2_ enhancing growth in some instances. Byssal attachment and condition index of adult *M. asperrima* decreased with elevated *p*CO_2_, while in contrast there was no effect on standard metabolic rate or pH*_e_.* The responses of larval and adult *M. asperrima* to elevated *p*CO_2_ measured in this study were more moderate than responses previously reported for intertidal oysters and mussels. Even this more moderate set of responses are still likely to reduce the abundance of *M. asperrima* and potentially other scallop species in the world’s oceans at predicted future *p*CO_2_ levels.

## Introduction

Ocean acidification has been called the “other CO_2_ problem”. Anthropogenic emissions of CO_2_ released into the atmosphere are being absorbed by the world’s oceans causing them to acidify [1], [2]. Compared with pre-industrial levels, the mean pH of the surface oceans has declined by more than 0.1 units [2]–[4] and assuming median emission scenarios, the Intergovernmental Panel on Climate Change [5], [6] has predicted pH will fall a further 0.3–0.5 units (pH 7.8–7.6) by 2100 and another 0.7–0.77 units (pH 7.4–7.43) by 2300 [1], [2], [4].

All evidence suggests that the decline in pH of the oceans will impact on marine ecosystems and organisms, and one of the most vulnerable phylum will be the calcifying molluscs [7]–[13]. Already along the northwest coast of the USA, oyster hatcheries have experienced mass mortalities from upwelling of deep acidified seawater onto the coasts [14]. Studies on adult molluscs including the oysters *Crassostrea gigas, Crassostrea virginica, Saccostrea glomerata*, [13], [15], [16] and mussels *Mytilus edulis* [17], [19], [20] have found decreased calcification and growth in response to elevated *p*CO_2_ [19], [20], perhaps because of the lowered saturation state of carbonate (CO_3_^2−^) and alterations to the organism’s acid-base status, which they attempt to compensate for by increasing their standard metabolic rate [21]–[23]. Studies on the responses of early life-history stages of molluscs to elevated *p*CO_2_ have found them to be even more sensitive than adult molluscs [9], [24]. Reduced fertilisation success, decreased larval size and development and increased larval abnormality and mortality have been found in the oysters *C. gigas, C. virginica, S. glomerata*, [11]–[13], [25]–[27] and mussels, *Mytilus galloprovincialis, Mytilus edulis* [18], [28]. Molluscs are not the only phylum affected by elevated *p*CO_2_, with negative responses also recorded for echinoderms, including the sea urchins *Hemicentrotus pulcherrimus*, *Enchinometra mathaei* [29], [30], *Paracetrotus lividus* [31], *Tripneustes gratilla, Pseudechinus huttoni, Evechinus chloroticus, Sterechinus neumayeri* [32], *Heliocidaris erythrogramma* [33]–[35] and the brittle star *Ophiothrix fragilis* [7].

Against this backdrop, recent studies have found that both echinoderms and molluscs have a capacity to acclimate to exposure of elevated *p*CO_2_ and fluctuations in pH [13], [36], [37]. Miller et al. [36] found that the larvae of the oyster *Crassostrea ariakensis* were not as adversely affected by the chronic exposure to high *p*CO_2_ as some other mollusc species. They hypothesised [36] that the increased resilience of the *C. ariakensis* to elevated *p*CO_2_ may be due to the exposure of this species to high levels of respiratory CO_2_ in the benthos [38]. Thomsen et al. [37] also found communities dominated by calcifying organisms, mostly mussels, thrived in naturally CO_2_ enriched waters of the Western Baltic Sea. When placed under artificially elevated *p*CO_2_ in the laboratory, this long term exposure to naturally elevated *p*CO_2_ increased the ability of *Mytilus* spp. to calcify. Similarly, Moulin et al. [31] found that echinoderms, *Paracentrotus lividus*, exposed to naturally fluctuating pH coped better with elevated *p*CO_2_ than those from a stable pH environment. It was found that fertilisation and larval development of *P. lividus* increased under elevated *p*CO_2_ when the parents were sourced from rock pools characterised by fluctuations in pH compared to adults that were sourced from rock pools where pH was more stable. Also, Parker et al. [13] found exposing adult estuarine oysters (*S. glomerata*) to elevated *p*CO_2_ during reproductive conditioning increased the resilience of their larvae. Larvae spawned from adults exposed to elevated *p*CO_2_ were larger and developed faster, but displayed similar survival compared with larvae spawned from adults exposed to ambient *p*CO_2_. In contrast, the subtidal echinoderms, *Strongylocentrotus droebachiensis* were less resilient when exposed to elevated *p*CO_2_ in the laboratory and less able to acclimatise over successive generations [39].

If long term fluctuating or stable exposure to elevated *p*CO_2_ increases the capacity of an organism to acclimate [31], [32], [37] then molluscs living in more stable pH environments, as found in some subtidal habitats, may be more susceptible to future elevated *p*CO_2_. Subtidal environments experience more stable physio-chemical properties than shallow subtidal or intertidal habitats where physio-chemical properties vary on a daily and seasonal basis [40].

Previous exposure to elevated *p*CO_2_ alone is unlikely to reliably predict the response of an organism to elevated *p*CO_2_. Mode of life and the capacity for ion regulation and maintenance of extracellular pH has been linked with responses of molluscs to elevated *p*CO_2_ [41]–[43]. The more tolerant taxa are characterised by high metabolic rates and high levels of mobility and activity [43]. For example, cephalopods, which experience high levels of haemolymph CO_2_ during bouts of physical exertion are able to increase calcification during exposure to elevated *p*CO_2_ [42], [44]. In contrast, bivalve and gastropod molluscs with a hypo-metabolic [13], [16], [43] mode of life and a decreased capacity to regulate ions and acid-base balance, typically show greater sensitivity than cephalopods to elevated *p*CO_2_. Some bivalves, such as the scallops (Pectinidae), are capable of swimming and during these swimming periods have elevated metabolism and oxygen consumption, often with 2 to 5 fold fluctuations in O_2_/CO_2_ gas exchange [45]. These short bouts of exercise, which result in an elevation of internal CO_2_ and subsequent acidification of the haemolymph, may provide scallops with a slightly increased capacity to cope with future external elevations in environmental *p*CO_2_ [43]. There is a strong link between the taxa that are tolerant to elevated *p*CO_2_ and/or organisms which experience metabolic fluctuations due to physical activity and higher metabolic rates [43]. Scallops are not as physiologically advanced as teleost fish or cephalopods [41]–[44], however, compared to other more sessile bivalves, their capacity for locomotion and metabolic fluctuations is pronounced [13], [16], [43], [44]. It may be that physiological fluctuations of internal *p*CO_2_ induced by physical activity, have enhanced the internal buffering systems of scallops, and in turn, this may enable them to better withstand external reductions of environmental pH.

To date, studies on the response of scallops to elevated CO_2_ have found reductions in calcification and larval growth in the scallop *Argopecten irradians* after 7 [46]and 36 days [47] of exposure. Also, larval development decreased, and abnormality increased when the scallop *Pecten maximus* was exposed to elevated *p*CO_2_ for 7 days [40]. In one of the first investigations on the physiological responses of scallops to elevated *p*CO_2_, Schalkhausser et al. [47] found that although there was no change in the standard metabolic rate of *P. maximus* when exposed to elevated *p*CO_2_, its ability to clap the upper and lower valves was reduced [48].

The aim of this study was to measure the responses of the scallop *M. asperrima* to elevated environmental *p*CO_2_. It was hypothesised that a subtidal species of scallop experiencing a stable physio-chemical environment and capable of swimming may have greater capacity to cope with elevated *p*CO_2_ compared to sessile molluscs such as oysters and mussels. Such increased capacity may be found in scallops that experience bouts of swimming which produce elevations of CO_2_ in the haemolymph and alterations in acid-base status. To test this hypothesis, the effects on both the larval and adult stages of the doughboy scallop *Mimachlamys asperrima*, were measured in response to elevated *p*CO_2_.

## Materials and Methods

### Broodstock Collection

*M. asperrima* is a commercially exploited subtidal bivalve endemic to south eastern Australia. Individuals grow to a shell length of 100 mm and remain a single sex throughout life [49]. *M. asperrima* is usually found in depths of 20–100 m. *M. asperrima* will attach to hard substrate using byssal threads, however, these threads can be easily dropped and the individual will swim away if threatened [49].

*M. asperrima* broodstock were collected by SCUBA from a depth of 20 m, approximately 500 m west of Honeymoon Bay (35.055434°S, 150.763794°E), on the eastern shore of Jervis Bay, NSW, Australia. As the collection site fell within Jervis Bay Marine Park, the relevant collection permit was acquired from NSW Department of Primary Industries. Following collection, scallops were placed in moist towels and hessian, packed in plastic tubs and transported to Port Stephens Fisheries Institute (PSFI). All seawater used in experimentation was collected from Little Beach (33.2°S, 152.11667°E), Nelson Bay, NSW, Australia and was filtered through 1 µm nominal filters (filtered sea water, FSW) prior to being used.

### Monitoring of CO_2_

In all early life experiments three elevated concentrations of *p*CO_2_ (600 µatm, 750 µatm, and 1000 µatm) (Table 1) and one ambient concentration (current atmospheric concentration of CO_2_: 390 µatm) were used being based on projections of the Intergovernmental Panel on Climate Change (IPCC) [5], [6] and the same as Parker et al. [12], [26]. These *p*CO_2_ levels correlated to a mean ambient pH_NBS_ of (mean ± SE = 8.20±0.02) and a mean elevated pH_NBS_ levels of (mean ± SE = 7.89±0.02, 7.81±0.02, 7.69±0.03), respectively. The pH of the seawater was manipulated to the chosen *p*CO_2_ concentration via direct bubbling of food grade CO_2_ in FSW while being mixed thoroughly. To determine the pH level corresponding to the *p*CO_2_ levels, total alkalinity (TA) was quantified at each water change (every two days) for all experiments using triplicate Gran-titration (Table 1, n = 3) [50], [51]. Following the titration, the TA and chosen *p*CO_2_ levels were entered into a CO_2_ system calculation program [52], using the dissociation constants of Mehrbach et al. [53], (Table 1) and the pH level corresponding with the desired *p*CO_2_ level was calculated. When the desired pH was reached the container (tightly capped jar or bucket depending on the experiment) was capped shut and there was very little change in pH over a 12 h period [12], [13], [26]. The pH was monitored using a pH electrode (WTW 3400*i*) calibrated daily using NBS buffers [54]. The pH of each replicate was checked three times daily and adjusted with additional direct bubbling of food grade CO_2_ where necessary.

**Table 1:** Mean recorded temperature, pH_NBS_, salinity and target *p*CO_2_ for each ontogenetic stage.

			Fertilisation and Trochophore		D-veliger		Umbonate veliger		Pediveliger		Spat	
Target *p*CO_2_ (µatm)	Temperature °C, (±SE)	Salinity psu (±SE)	Mean Total Alkalinity (±SE) µmol kg^−1^	Mean pH_(NBS)_ (±SE)	Mean Total Alkalinity (±SE) µmol kg^−1^	pH _NBS_ (±SE)	Mean Total Alkalinity (±SE) µmol kg^−1^	pH _NBS_ (±SE)	Mean Total Alkalinity (±SE) µmol kg^−1^	pH _NBS_ (±SE)	Mean Total Alkalinity (±SE) µmol kg^−1^	pH_ NBS_ (±SE)
390	20 (0.5)	33.3 (0.4)	2340 (44)	8.20 (0.02)	2321 (36)	8.19 (0.02)	2329 (45)	8.19 (0.02)	2335 (44)	8.19 (0.02)	2333 (42)	8.19 (0.02)
600	20 (0.5)	33.2 (0.4)		7.89 (0.02)		7.89 (0.02)		7.89 (0.03)		7.89 (0.02)		7.89 (0.02)
750	20 (0.5)	33.3 (0.3)		7.81 (0.03)		7.81 (0.02)		7.81 (0.02)		7.81 (0.03)		7.81 (0.02)
1000	20 (0.5)	33.3 (0.3)		7.69 (0.02)		7.69 (0.03)		7.69 (0.02)		7.69 (0.03)		7.69 (0.02)

Target *p*CO_2_ calculated from the Total Alkalinity (TA), of the four treatment levels, for each ontogentic stage; fertilisation, trochophore, D-veliger, umbonate veliger, pediveliger and spat experiments. There were 4 replicates for each treatment for the fertilisation, trochophore and D-veliger; and 3 replicates per treatment for umbonate, pediveliger and spat experiments. pH values corresponding with target *p*CO_2_ were provided by the Lewis and Wallace [52] calculation program using the dissociation constants of Mehrbach [53]. Temperature and salinity remained constant throughout the experiment. The measurements of pH were taken in each replicate container at each water change, n = 4 (fertilisation and trochophore experiment), n = 12 (D-veliger experiment) n = 9 (umbonate and spat experiment), n = 6 (pediveliger experiment).

### Fertilisation and Early Development

To determine the effect of acute exposure to elevated *p*CO_2_ on fertilisation after 2 h and early development after 24 h, spawning of adult *M. asperrima* was induced by injection of 0.05 ml 1×10^−2^ M serotonin solution into ten females and five male scallops through multiple injections into the gonad and one injection into the adductor muscle [55]–[57]. Serotonin solution was made by dissolving creatine sulphate complex (C_14_H_21_N_5_O_6_S H_2_O; Merck, Damnstdat, Germany) in filtered sea water [55]–[57]. Following injections, each scallop was isolated in a 400 mL container of FSW and observed.

Eggs from five females were collected from the containers, mixed in equal proportions and stocked in 5 L glass jars at a density of 16 eggs mL^−1^. For each *p*CO_2_ level (390 µatm, pH 8.2; 600 µatm, pH 7.89; 750 µatm, pH 7.81; and 1000 µatm, pH 7.69), 20 L containers of FSW were adjusted to the appropriate pH corresponding to the three levels of elevated *p*CO_2_ by direct bubbling of CO_2_. Once equilibrated this water was distributed among replicate 5 L jars, which were then tightly capped shut. There were 4 replicate jars per *p*CO_2_ level. Sperm was collected and pooled in equal volumes from five males. To determine the correct sperm quantity, a 1 mL subsample of egg solution was taken. The sperm solution was then added incrementally to these eggs in known volumes and observed microscopically (*Leica* 100x). When enough sperm was added for there to be one active sperm visible per egg the volume of sperm was recorded. The volume of sperm to provide the equivalent concentration as the subsample was then added to all replicate jars. Two hours after sperm addition each replicate jar was thoroughly mixed and a 10 mL sample was taken. To each sample, 1 mL of 10% formalin was added to prevent further embryo development. Each sample was then passed through a 20 µm mesh screen and the contents were resuspended to a 1 mL sample. The percentage fertilisation for each sample was then determined under a compound microscope (*Leica* 100x) on a Sedgewick-Rafter counting slide. The first 30 eggs encountered were examined for signs of fertilisation. Fertilisation was defined as when a cleavage plane through the egg could be distinguished [12]. Data were expressed as a percentage fertilised per replicate. Twenty four hours after sperm addition, another 10 mL sample was taken from each replicate jar and filtered using the method above. The first 30 embryos encountered within each sample were examined and were deemed to be either fully developed trochophores or “other”. Trochophores were defined as ciliated and motile [58], [59] and “other” was primarily composed of embryos which had arrested development and unfertilised eggs. This was then expressed as percentage trochophore at 24 h (Table 2).

**Table 2:** Summary of experimental design for each ontogenetic stage.

	Fertilsation & Trochophore	D - veliger	Umbonate veliger	Pediveliger	Spat
Age at beginning of exposure	0	4 days	10 days	16 days	30 days
No. Replicates	4	4	3	3	3
Density mL^−1^	16	5	2.5	2.5	0.006
Container volume/type	5 L jar	10 L bucket	10 L bucket	10 L bucket	10 L bucket
Time exposed	2, 24 h	4, 6 days	4, 6 days	4, 6 days	6 days

### Larval Development

To determine the effect of elevated *p*CO_2_ on larval development, adult *M. asperrima* were induced to spawn using the technique of temperature shock [57], [60], [61]. This technique was used because it yields a larger number of gametes, and serotonin injection does not provide the numbers of eggs and sperm required for this experiment. The temperature shock technique involved placing ten males and ten female scallops haphazardly selected from the broodstock population in a bath of FSW at 24°C (4°C warmer than the ambient temperature of 20°C). Following spawning, gametes were collected separately to avoid contamination and returned to individual containers of FSW at ambient temperature (20°C). Collected eggs from at least 20 females (15 million) were added to a 20 L bucket. Sperm was collected from at least 10 males and added to eggs at a concentration of 825 mL^−1^ (1∶1.1 egg to sperm ratio, sperm concentration determined using a haemocytometer under a light microscope; *Leica* 100x) and mixed thoroughly, once sufficient fertilisation was observed microscopically (90% of eggs showing cleavage, about 1 h post sperm addition *Leica* 100x). Gametes were stocked in a 1000 L polyethylene tank filled with FSW (390 µatm; pH 8.20; 20°C; salinity 34.5 psu). They were then reared as a single batch using the methods of O’Connor et al. [55] and transferred to experimental conditions at 3 stages; D-veliger (4d), umbonate veliger (10 d), and pediveliger (16 d). Larvae were fed twice daily on a known algal diet consisting of equal parts Tahitian *Isochrysis aff. galbana, Pavlova lutheri* and *Chaetoceros calcitrans*. Algal concentrations ranged according to larval size, an equivalent of 3500 T. *Isochrysis* cells larva^−1^ day^−1^ was fed at the D-veliger stage. This incrementally increased to 20,000 T. *Isochrysis* cells larva^−1^ day^−1^ at the pediveliger stage [62], [63].

Once larvae reached the beginning of each ontogenetic stage (D-veliger, 4 d; umbonate veliger, 10 d; and pediveliger, 16 d), they were sieved through a 45, 90 and 130 µm mesh respectively. Larvae retained were then counted and added to 10 L replicate buckets in each experiment. The larval density at the beginning of each experiment was 5 mL^−1^ for D-veligers, and 2.5 mL^−1^ for umbonate and pediveliger stages. Larvae were placed in replicate 10 L buckets filled with 8 L of FSW and were maintained at a constant 20°C. Buckets were capped shut at all times. Each bucket had a small hole in the lid through which air was very gently pumped in by air hose to promote some water movement. There were four replicate buckets for D-veliger experiments and three replicate buckets for the umbonate, and pediveliger experiments at each *p*CO_2_ level (390 µatm, pH 8.2; 600 µatm, pH 7.89; 750 µatm, pH 7.81; and 1000 µatm, pH 7.69;Table 1, 2). The pH of each container was measured 3 times daily, and if necessary, the pH was adjusted with additional CO_2_. Every two days the seawater in each replicate bucket was changed. New replicate buckets were cleaned using Virkon S solution (Antec Corp, North Bend, WA, USA) rinsed with new FSW, and allowed to air dry. They were then filled with FSW of the same temperature and adjusted to the appropriate pH level. Each experimental bucket was sieved through 45 µm mesh screen and larvae were captured. The larvae were then transferred to their new replicate bucket set at the correct *p*CO_2_ level and capped shut. For each larval stage, after four days of exposure the contents of each replicate bucket were stirred until homogenised and 500 mL of water was collected. Fifty mL of 10% buffered formalin was added to each collected sample. The first 30 larvae encountered were measured for shell length along their antero-posterior axis. This was done on a Sedgewick-Rafter slide under a compound microscope (*Leica* 100x) using an ocular micrometer. This collection and measurement was then repeated at six days of exposure.

### Settlement and Exposure of Spat

To collect spat, settlement surfaces (balls of plastic netting) were suspended in the 1000 L main culture tank (originally stocked with larvae) 20 days after fertilisation. Spat were collected by rinsing these settlement surfaces with seawater over a mesh screen [55]. All spat removed from the screen were counted by eye and separated into 12 groups of 50. Spat were then added to a small purpose built “spat screen”. There were 50 spat per spat screen. Each spat screen was circular and 100 mm in diameter with raised plastic sides and a 130 µm mesh base, that prevented the spat from passing through. During experimental conditions, spat were fed twice daily on a known algal diet consisting of equal parts Tahitian *Isochrysis aff. galbana, Pavlova lutheri* and *Chaetoceros calcitrans*, at a concentration equivalent to 20,000 T. *Isochrysis* cells larva^−1^ day^−1^. To determine the effect of elevated *p*CO_2_ on the growth of spat, spat were exposed to the experimental levels of *p*CO_2_ (µatm, pH 8.2; 600 µatm, pH 7.89; 750 µatm, pH 7.81; and 1000 µatm, pH 7.69 Table 1, 2) for 6 days. The bucket set up and pH manipulation remained the same as per larval experiments. There were three 10 L replicate buckets at each *p*CO_2_ level, one “spat screen” each containing 50 spat was added to each replicate bucket. Every two days the culture seawater was changed, at the time of each water change, each spat screen was individually removed from the experimental bucket and rinsed gently with FSW set at the experimental *p*CO_2_ level to remove any bacteria or algae that had settled on the screen. The “spat screen” was then gently placed into the new replicate bucket of corresponding *p*CO_2_ adjusted FSW (Table 2). The pH of each replicate bucket was monitored 3 times per day and adjusted where necessary. After 6 days exposure, the shell length (antero-posterior measurement) of 30 randomly selected spat were measured from each replicate bucket using a Sedgewick-Rafter slide under a compound microscope (*Leica* 100x) with an ocular micrometer.

### Physiological Measurements on Adults

To determine the effect of elevated *p*CO_2_ on adult physiology, 60 male and 60 female broodstock individuals were randomly selected. From this pool, 30 males and 30 females were randomly selected and assigned to the elevated *p*CO_2_ and ambient *p*CO_2_ treatment. The ambient level was the current atmospheric *p*CO_2_ level of 390 µatm, mean pH_NBS_ (±SE) 8.20 (±0.02). The elevated level used was that predicted for the year 2100, 750 µatm, mean pH_NBS_ (±SE) 7.81 (±0.03) [5], [6]. The correct pH level for the elevated *p*CO_2_ treatment was recalculated every water change (every 2 days) following triplicate gran titration. Following the titration, the total alkalinity (TA) and selected *p*CO_2_ level were entered into a CO_2_ system calculation program [52], using the dissociation constants of Mehrbach et al. [53] to determine the correspong pH value for the desired *p*CO_2_ level. There were 3 replicate 200 L tanks for each the two *p*CO_2_ levels, each tank was stocked with 20 scallops (10 male, 10 female). Scallops were housed mid water in large mesh bags for 5 weeks and water temperature was maintained at 20°C, each tank was filled with FSW. To ensure that there were no *a-priori* differences in sizes of scallops among groups, shell lengths (antero-posterior measurement) and whole weight of 15 randomly selected individuals from each of the ambient and elevated treatments were measured and analysed using ANOVA (Shell length: F_(1, 28)_ = 0.68 P = >0.05, Whole weight: F_(1, 28)_ = 0.29 P = >0.05).

The elevated level of *p*CO_2_ was maintained using pH negative feedback systems (Aqua Medic, Aqacenta Pty Ltd, Kingsgrove, NSW, Australia; accuracy ± 0.01 pH units). Food grade CO_2_ was bubbled directly into the tanks containing adult individuals via a CO_2_ reactor to ensure proper mixing and in turn, reduce pH. A pH probe connected to a controlling computer was placed within each tank (probes were calibrated weekly using NBS buffers). When the desired pH level was reached, the delivery of CO_2_ was automatically stopped by a computer signal to a solenoid valve. Each tank set to elevated *p*CO_2_ was controlled by its own independent pH controlling system. The pH values of each tank were monitored daily, and the pH electrode of each controlling system was checked against another calibrated pH probe (NBS buffers, WTW 3400*i*) daily. Every second day of the five weeks of exposure, each tank received a complete water change. New FSW was pumped into identical tanks at least 24 h prior to the scheduled change, the CO_2_ monitoring systems were also transferred to the new tanks prior to the water change. This allowed the new FSW to be at the same temperature, and CO_2_ level as the previous FSW. It also allowed for scallops to be out of the water only briefly during the water change. Following the water change, each old tank was cleaned vigorously using Virkon S solution (Antec Corp, North Bend, WA, USA), and then rinsed with fresh water and allowed to air dry prior to re-filling. At each water change, dead scallops (if any) were removed, recorded and replaced with a scallop of the same sex where possible; replacement scallops were isolated in each tank from the original stocking population to prevent them being mistaken as part of the original stocked scallops. At every water change the number of scallops byssally attached to the mesh bag or another scallop’s shell in each replicate from the original stocked scallops (not replacements) was also recorded.

In weeks 2, 4 and 5 of experimental conditions, a male and female scallop from the original stocked scallops of each tank was selected at random for adult measurements. These measurements included Standard Metabolic Rate (SMR), and Haemolymph pH (pH_e_) and O_2_ (*p_e_*O_2_ hPa) levels. After 5 weeks exposure, each scallop from the original stocking (not replacements) was removed by slicing their byssal threads with a scalpel. The number of threads severed for each scallop was recorded. Adults were fed each day an algal mixture consisting of 50% *Chaetoceros muelleri* and 50% T. *Isochrysis* at a concentration equivalent to 2×10^9^ T. *Isochrysis* cells scallop^−1^ d^−1^ [62].

### Standard Metabolic Rate (SMR)

SMR was determined using the closed respiratory system method of Parker et al. [13]. At each sampling time (weeks 2, 4 and 5), two scallops (one male, one female) were randomly selected from each tank for measurements (total 12; 6 from 390 µatm, 6 from 750 µatm). Scallops were placed in individual 500 mL airtight chambers filled with FSW at the correct *p*CO_2_ level. Each chamber was fitted with a fibre-optic O_2_ probe (PreSens dipping probe DP-PSt3, AS1 Ltd, Palmerston North, New Zealand). When scallops were placed in the chambers there were no air spaces which may interfere with the O_2_ readings. The probes were calibrated using a two-point calibration (0% and 100%) and all measurements were done at the experimental temperature of 20°C. The time taken to reduce the percentage oxygen saturation of seawater in the chamber from 100% to 80% was recorded. Time was only recorded when scallops were actively respiring (time that oxygen levels were decreasing). Prior to these SMR measurements, feeding had ceased for 24 h to remove any variability associated with digestive metabolism. Following the measurements, scallops were removed from the chambers and the dry tissue mass (tissue removed and dried in an oven at 80°C for 72 h) was measured. SMR was calculated for each scallop using the following equation (1):
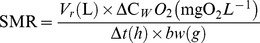



#### Equation 1. SMR calculation

Where: SMR is oxygen consumption normalised to 1g of dry tissue mass (mg O_2_ g^−1^ dry tissue mass h^−1^), *V_r_* is the volume of the respiratory chamber minus the volume of the scallop (L), ΔC_W_O_2_ is the change in water oxygen concentration measured (mg O_2_L^−1^), Δ*t* is the measuring time (h), bw is the dry tissue mass (g) [13].

### Condition Index

The condition index of scallops was determined at weeks 2, 4 and 5, from the individuals used for SMR measurements (total 12; 6 from 390 µatm, 6 from 750 µatm). Condition Index was defined as a ratio of tissue weight to shell weight (as a proxy for shell internal volume). Scallop tissue and shells were dried in an oven for 72 h at 80°C, and weighed separately. Condition index was calculated for each individual as in Equation 2.
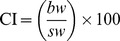



#### Equation 2. Condition Index calculation

Where: CI  =  condition index, bw is the dry tissue mass (g), sw is the dry shell mass (g).

### Haemolymph pH (pH_e_) and O_2_ (*p*_e_O_2_)

To determine haemolymph pH (pH_e_) and O_2_ (*p_e_*O_2_ hPa) approximately 0.5 mL of haemolymph fluid was extracted from the scallop’s adductor muscle using a 1 mL needled syringe. This was done on the scallops after they had their SMR analysed (total 12; 6 from 390 µatm, 6 from 750 µatm). While each scallop was held open, a 1 mL needled syringe was placed in the adductor muscle and haemolymph slowly extracted. This was done to prevent any air bubbles from passing into the haemolymph sample. The haemolymph was immediately placed into a 2 mL centrifuge vial where it was analysed for O_2_ content. To measure O_2_ content, a fibre-optic probe (PreSens dipping probe DP-PSt3, AS1 Ltd, Palmerston North, New Zealand) was placed in the haemolymph and O_2_ levels recorded (*p_e_*O_2_ hPa). Following this measurement, haemolymph pH was measured using a fine pH electrode (Metrohm 826 pH mobile). The pH electrode was calibrated for each use with NBS buffers.

### Data Analysis

To determine differences among *p*CO_2_ treatments for shell length of D-veliger and umbonate veliger larvae, as well as physiological measurements of adults (SMR, condition index, and haemolymph pH and O_2_), data were analysed using a three factor analysis of variance. “Time” and “*p*CO_2_” were fixed factors and “Tank” (for adult measurements) or “Container” (for larval experiments) was nested in *p*CO_2_. To determine any differences in shell length of pediveligers and spat among *p*CO_2_ treatments data were analysed using a two factor analysis of variance where “*p*CO_2_” was fixed and orthogonal and “Container” was nested in *p*CO_2_. In all analysis with “Tank/Container” as a factor, if the “Tank/Container” factor was not significant (using α >0.25 to guard against increased possibility of Type 1 statistical error [64]), replicates were pooled for this factor and the analysis redone without “Tank/Container” as a factor. To determine any differences in means of percentage fertilisation, development to trochophore, and the number of byssal threads per scallop were analysed using a one factor analysis of variance with *p*CO_2_ as the fixed factor. Spat growth from beginning (t_0_) to six days treatment, and adult exposure starting shell length and whole weight were analysed using a one factor analysis of variance where shell length or whole weight was the fixed factor. The program GMAV 5 for Windows was used for all analyses of variance, heterogeneity of variances was assessed prior to analysis using Cochran’s test, but no significant heterogeneities among variances were detected [64]. Differences in the means were detected using a series of Student Newman Kuels (SNK test) on each parameter [65]. Planned comparisons were made to assess differences between extremes of treatment gradients. Linear regression analysis was used to determine the significance of the slope for both *p*CO_2_ treatments when assessing the percentage of individuals byssally attached over time. For the linear regression analysis *p*CO_2_ treatment was the dependent variable and days exposed was the independent variable.

## Results

### Fertilisation and Early Development

**Figure 1:**
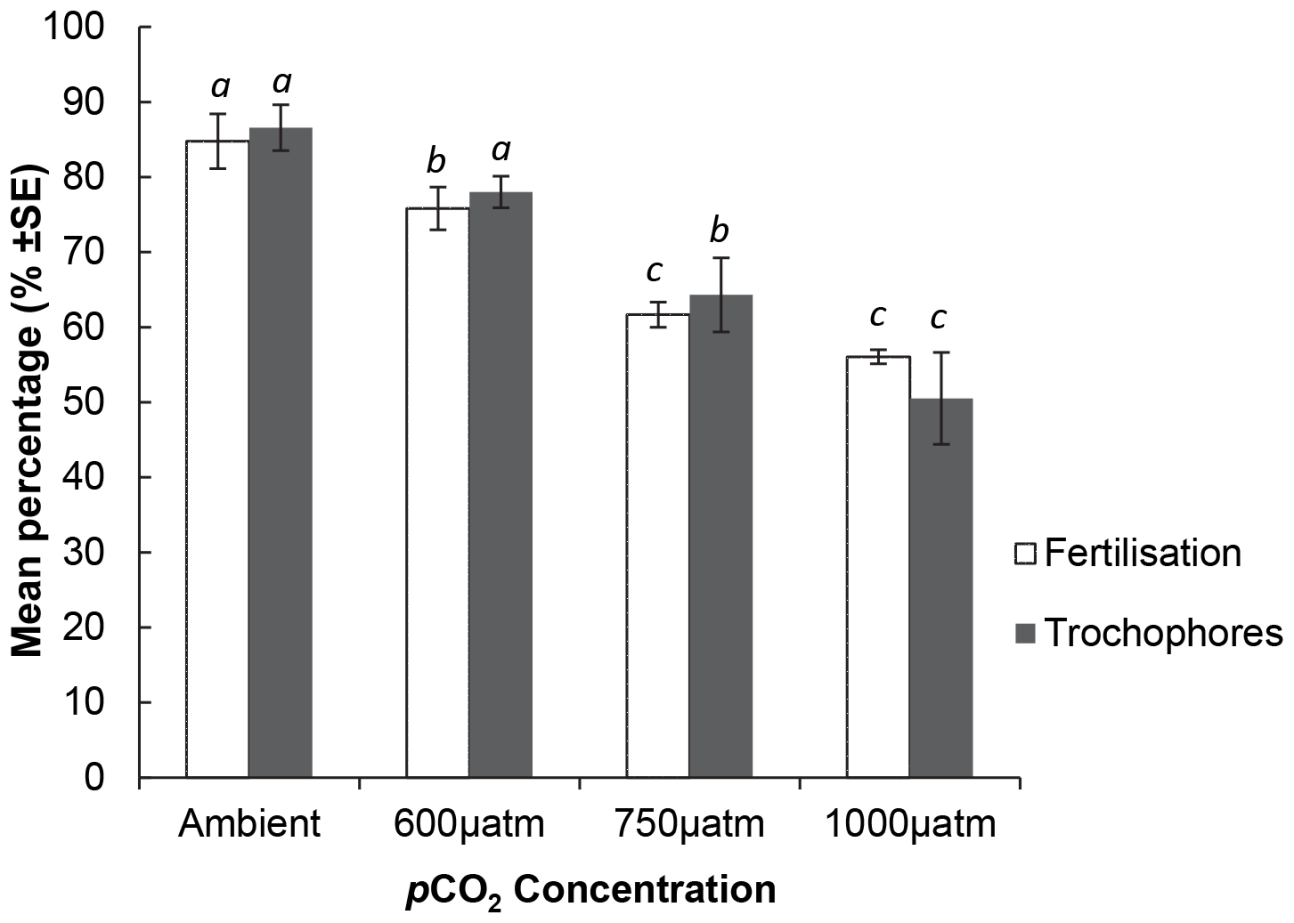
The effect of elevated *p*CO_2_ on the fertilisation and early development of *M. asperrima* gametes. Mean percentage of fertilisation and development to trochophore of *M. asperrima* among ambient and elevated *p*CO_2_ levels (390 µatm, 600 µatm, 750 µatm, 1000 µatm) ±MSE, n = 4. Letters above columns represent results of SNK tests. Different letters represent significant differences detected among CO_2_ treatments, corresponding letters represent no significant differences among treatments.

There was a significant effect of elevated *p*CO_2_ on fertilisation success, and percentage development to trochophore larvae (Figure 1; Table 3). As the *p*CO_2_ concentration increased, fertilisation decreased. Post-hoc analysis using SNK tests found the percentage of fertilisation in the ambient treatment (mean 85%) was significantly greater than all other treatments. The percentage fertilisation at 600 µatm (mean 75%) was significantly greater than the 750 and 1000 µatm treatments which were not significantly different from each other (Figure 1). Similarly, as *p*CO_2_ concentration increased the percentage of embryos reaching the trochophore stage significantly decreased, with only about 50% of embryos developing to trochophore at 1000 µatm. Post-hoc analysis using SNK tests found there was no significant difference in the percentage of trochophores between ambient (mean 85%) and 600 µatm, (mean 75%) treatments. There was, however, a significantly lower percentage of embryos to reach the trochophore stage at 750 µatm (mean 65%), which was significantly greater than 1000 µatm treatment (mean 50%) (Figure 1, Table 3).

**Table 3:** Summary of analysis of variance on *M. asperrima* early life history exposure to elevated *p*CO_2_.

		Fertilisation			% Trochophore			D-veliger shell length			Umbonate veliger shell length (4,6d)			Pediveliger shell length			Spat shell length		
Source of variation	*df*	*MS*	*F*	*P*	*MS*	*F*	*P*	*MS*	*F*	*P*	*MS*	*F*	*P*	*MS*	*F*	*P*	*MS*	*F*	*P*
Time	1							21842464	18.7421.55	***	1267	41	***						
*p*CO_2_	3	687	27	***	1001	13.2	***	22662197	36.425.49	***	259	1.9	ns	4491	12.6	**	23591	1.54	ns
Container/Tank x *p*CO_2_	12							62	1.6634	ns	136	2.5	*	358	1.67	ns	15339	1.14	ns
Time x *p*CO_2_	3							13420	1.161.05	ns	31.2	1	ns						
Time x Container/Tank (*p*CO_2_)	12							1164	3.102.48	*	31.1	0.6	ns						

Summary of analysis of variance of the means of % fertilisation and % development to trochophore of *M. asperrima* embryos in response to 2 and 4 h elevated *p*CO_2_ (390, 600, 750, 1000 µatm), and the shell length of D-Veliger, umbonate, pediveliger larvae and spat of *M. asperrima* after 4 and 6 days in response to exposure to elevated *p*CO_2_ (390, 600, 750, 1000 µatm). Significance level is indicated by asterisks, * *P*<0.05; ** *P*<0.01; *** *P*<0.001. Percentage fertilisation and trochophore development was analysed using a single factor ANOVA with “*p*CO_2_” as the fixed factor. D-veliger and umbonate shell length was a 3 way analysis where “Time” and “*p*CO_2_” were fixed factors and “Container/Tank” was nested in *p*CO_2_. Pediveliger and spat shell length were analysed using a 2 factor ANOVA with the “Container/Tank” factor nested in “*p*CO_2_”.

### Larval Development in Acute Exposure Experiments

**Figure 2:**
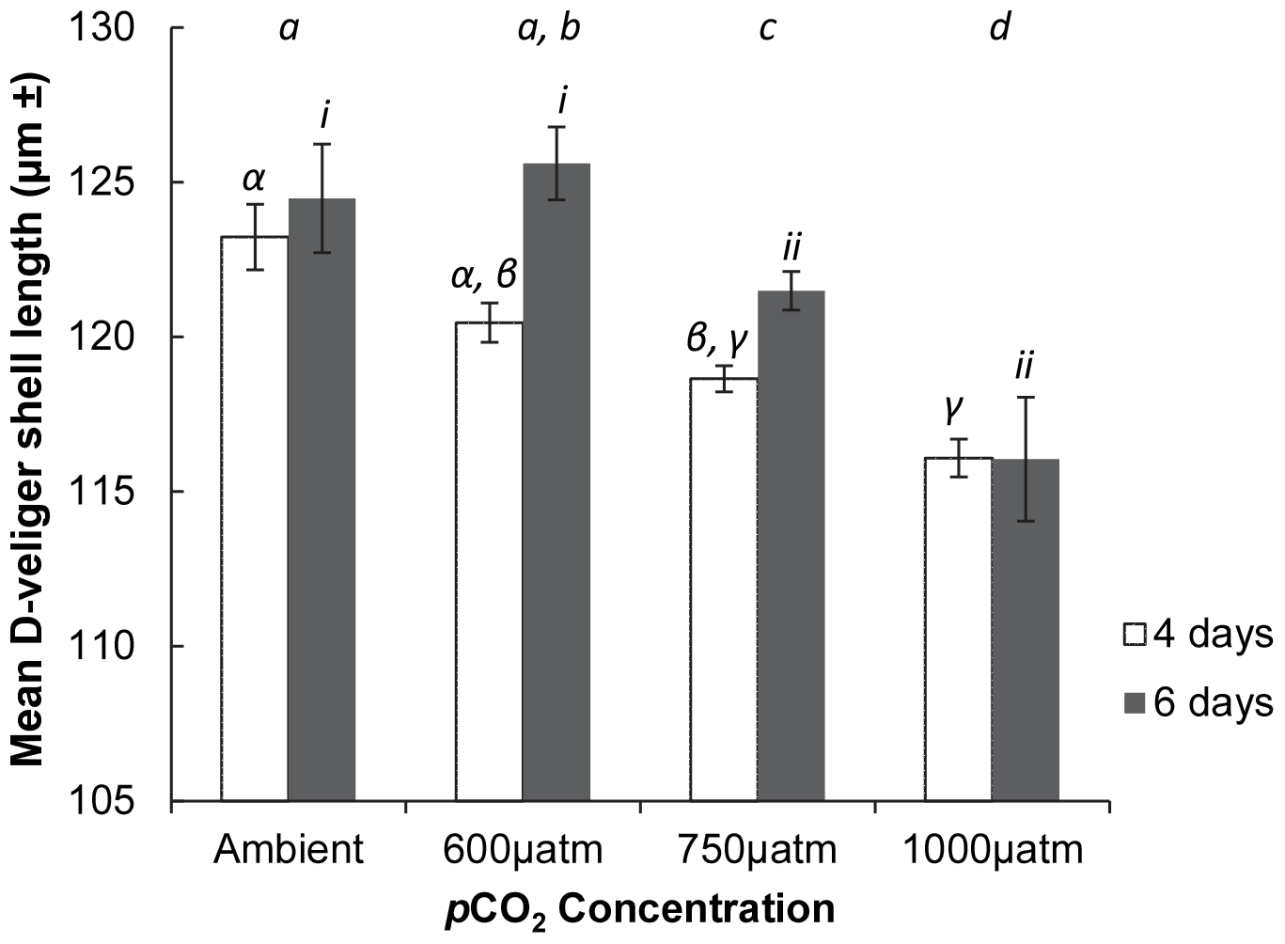
The effects of elevated *p*CO_2_ on *M. asperrima* D-veliger shell length. Mean shell length of *M. asperrima* D-veliger larvae following 4 and 6 days treatment at ambient and elevated *p*CO_2_ levels (390 µatm, 600 µatm, 750 µatm, 1000 µatm) ±MSE, n = 4. Letters above columns represent results of SNK tests, different letters represent significant differences detected, corresponding letters represent no significant differences detected among those treatments. Roman numerals *i – ii* represent differences among CO_2_ treatments after 4 days, Greek letters α – β represent differences among CO_2_ treatments after 6 days and Latin letters *a –c* represent SNK results of overall mean larval shell length disregarding sampling times.

**Figure 3:**
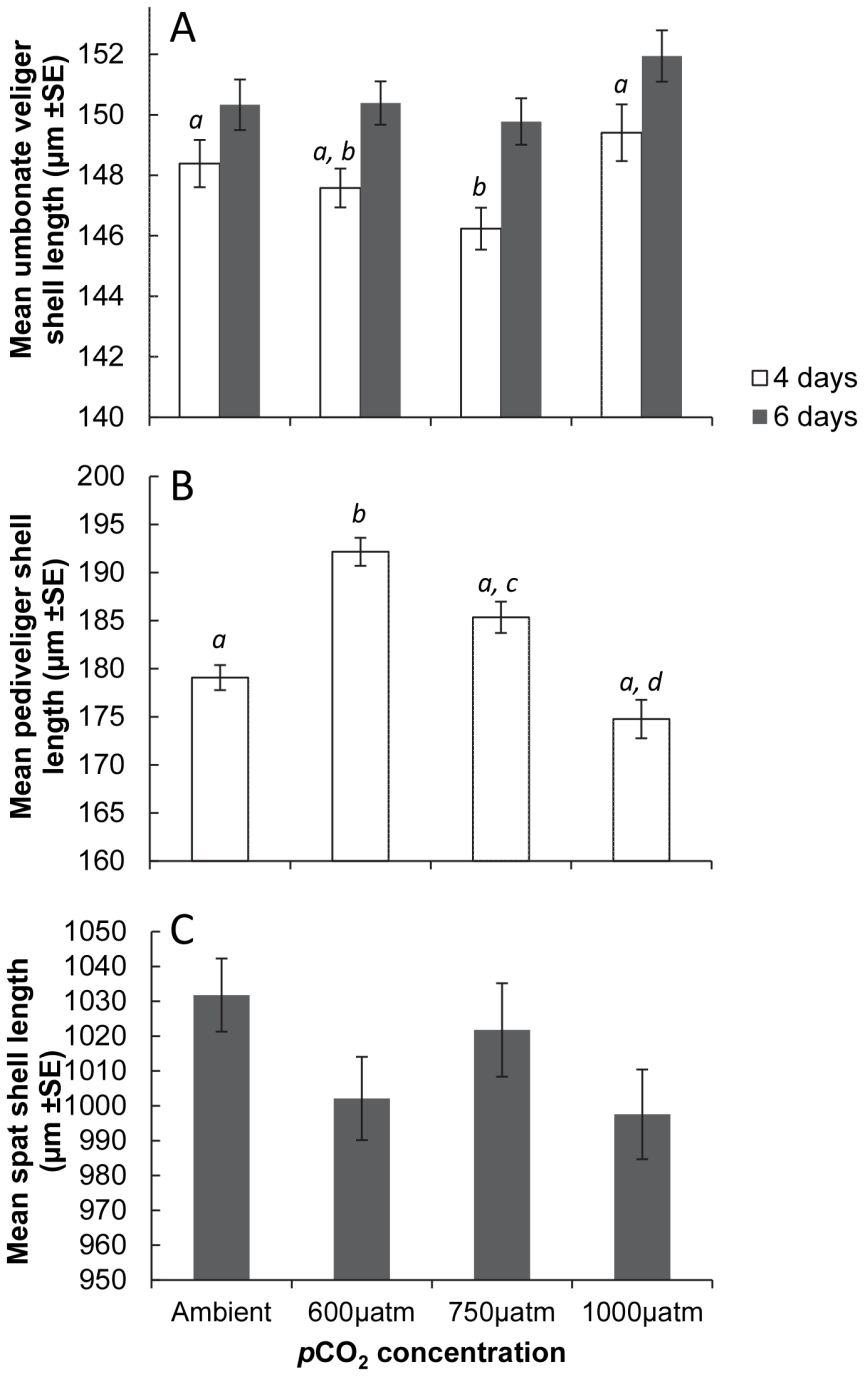
The effects of elevated *p*CO_2_ on *M. asperrima* Umbonate veliger, pediveliger and spat shell length. Mean shell length (antero-posterior axis) following exposure to Ambient, and elevated (600, 750, 1000 µatm) levels of *p*CO_2_. A) Umbonate larvae shell length following 4 (White) and 6 (grey) days exposure. B) Pediveliger larvae shell length following 4 days exposure. C) Spat shell length following 6 days exposure. ±MSE, n = 3. Letters above columns represent results of SNK tests, different letters represent significant differences detected, corresponding letters represent no significant differences detected among those treatments. Letters *a - c* represent differences in shell length among CO_2_ treatments for umbonate veligers (A) and pediveligers (B) following 4 d exposure. No significant differences in shell length were detected among treatments for umbonate veligers (A) and spat (C) following 6 d exposure.

In general, as *p*CO_2_ increased, the size of D-veliger larvae decreased (Figure 2; Table 3). After four days, the shell length of D-veligers in ambient and 600 µatm conditions were significantly greater than the 750 and 1000 µatm treatments (Figure 2; Table 3). After six days of exposure the shell length of D-veligers in ambient conditions (mean ± SE = 124±1.7 µm n = 4 and in the 600 µatm (mean ± SE = 125±1.1 µm n = 4) treatment was significantly greater than D-veligers in the 750 µatm (mean ± SE = 121 µm±0.6 n = 4) and 1000 µatm (mean ± SE = 116 µm±1.9 n = 4) treatments, which were not significantly different from each other. There was no overall significant effect of elevated *p*CO_2_ on the shell growth of umbonate veligers, however, post hoc analysis found that after 4 d exposure umbonate veligers in the 750 µatm treatment were significantly smaller than in the ambient and 1000 µatm treatments (Figure 3A; Table 3). Following 6 d exposure there were no significant differences in shell length of umbonates among treatments (Figure 3A; Table 3). There was a significant effect of elevated *p*CO_2_ on the shell length of pediveligers, but in contrast to the trend for decreased shell length with increased *p*CO_2_, following six days exposure, shell length was significantly greater in the 600 µatm treatment (Figure 3B; Table 3). During the pediveliger experiment, after 4 d many of the larvae in the control treatments died and the experiment was halted, explaining the absence of pediveliger data on day 6. The mean shell length of spat was greatest in the control treatment (mean ± SE = 1031 µm±10.4 n = 3), and the 1000 µatm treatment had the smallest mean spat shell length (mean ± SE = 997 µm±12.8 n = 3) this was, however, not a significant difference (Figure 3C; Table 3).

### Adults

**Figure 4:**
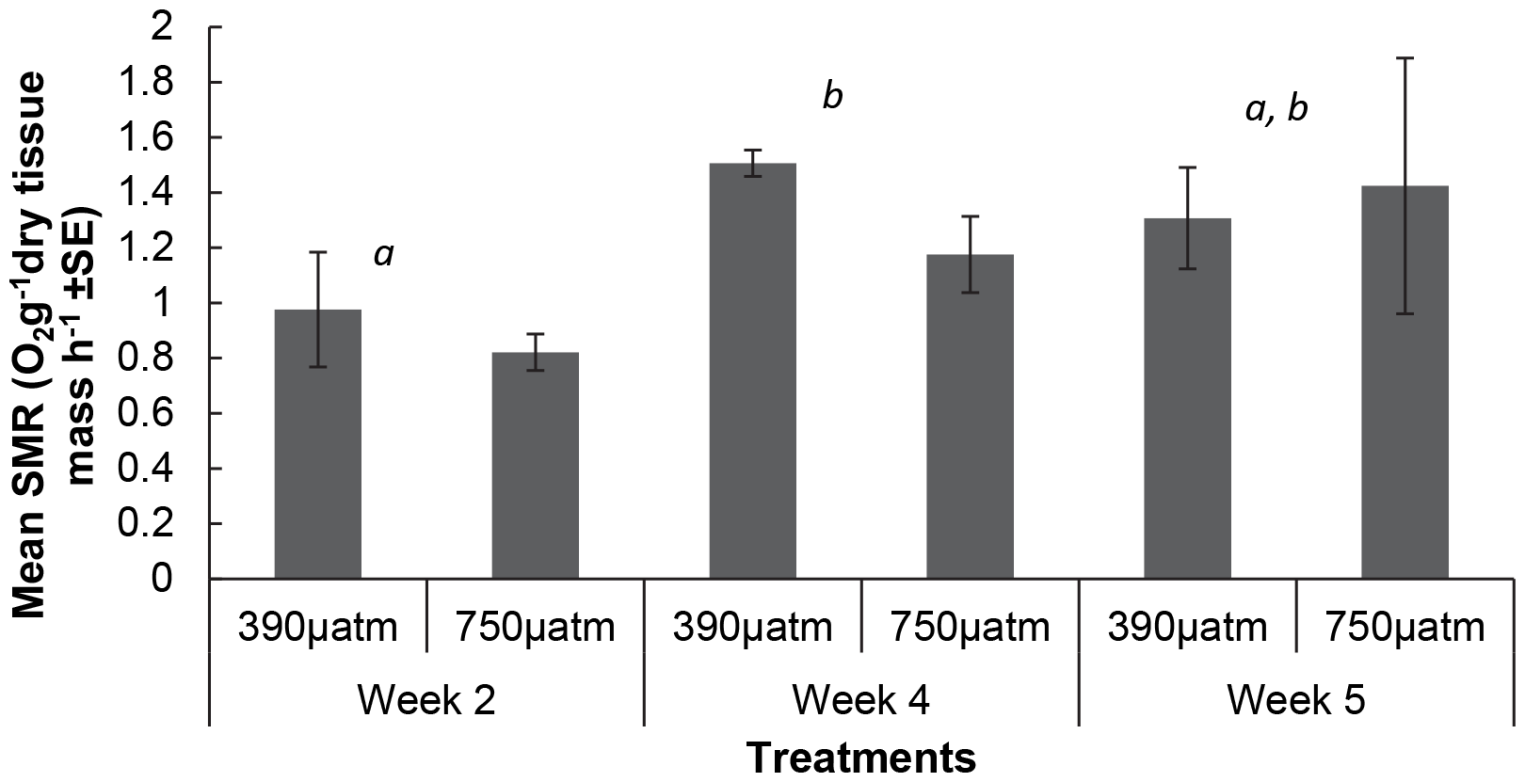
The mean of the standard metabolic rate (SMR) of *M. asperrima* in response to ambient and elevated *p*CO_2_. Mean SMR after exposure to ambient and elevated *p*CO_2_ (390 µatm, 750 µatm), for 2, 4 and 5 weeks ±MSE, n = 3. Letters above columns represent results of SNK tests, different letters represent significant differences detected among sampling times disregarding CO_2_ treatment.

**Figure 5:**
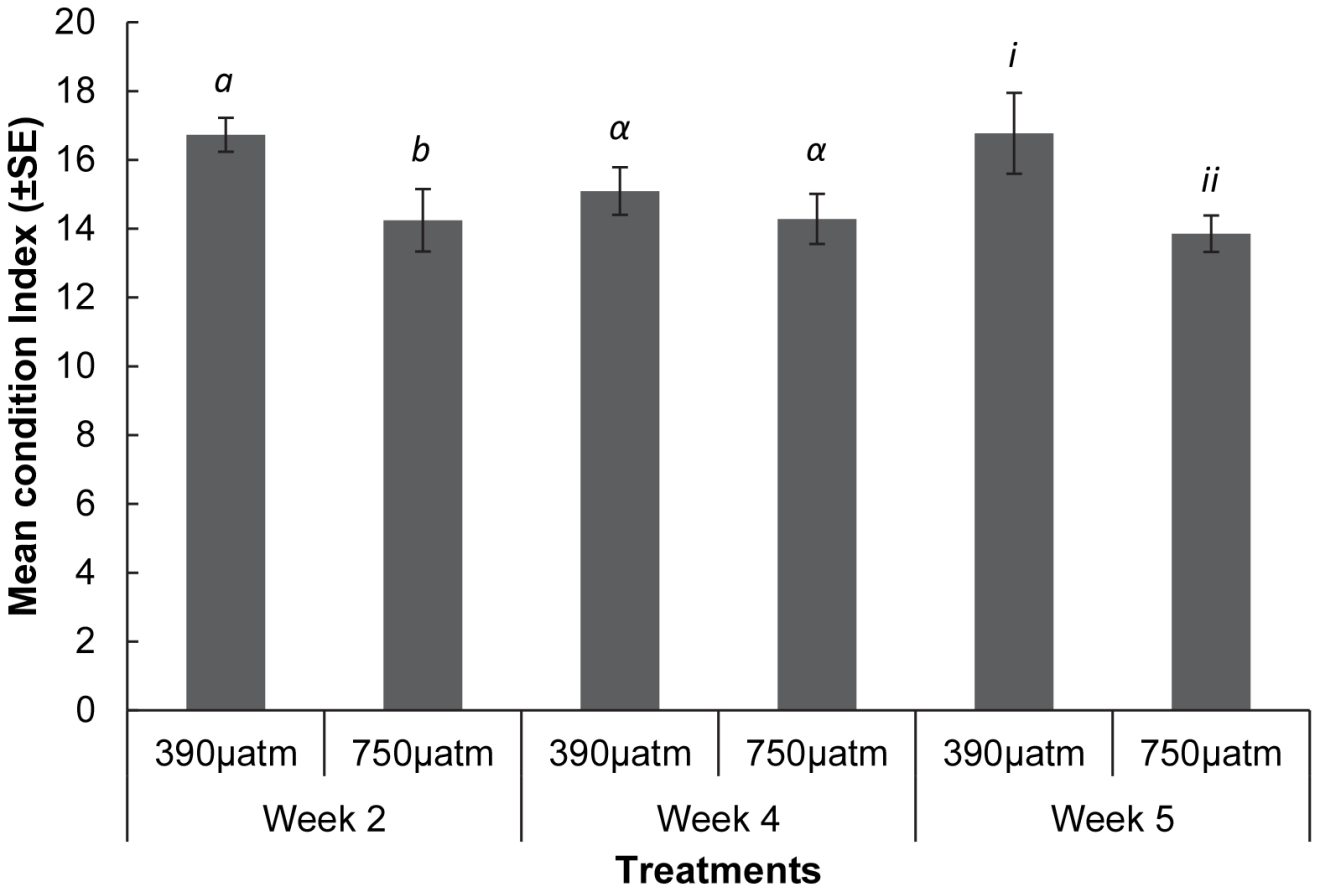
The mean condition index of *M. asperrima* individual*s* in response to ambient and elevated *p*CO_2_. Mean condition index after exposure to ambient and elevated *p*CO_2_ (390 µatm, 750 µatm), for 2, 4 and 5 weeks ±MSE, n = 3. Symbols above columns represent results of SNK tests, Different Latin *a - b*, Greek α - α, and Roman *i - ii* symbols represent differences within each sampling time between *p*CO_2_ treatments.

**Figure 6:**
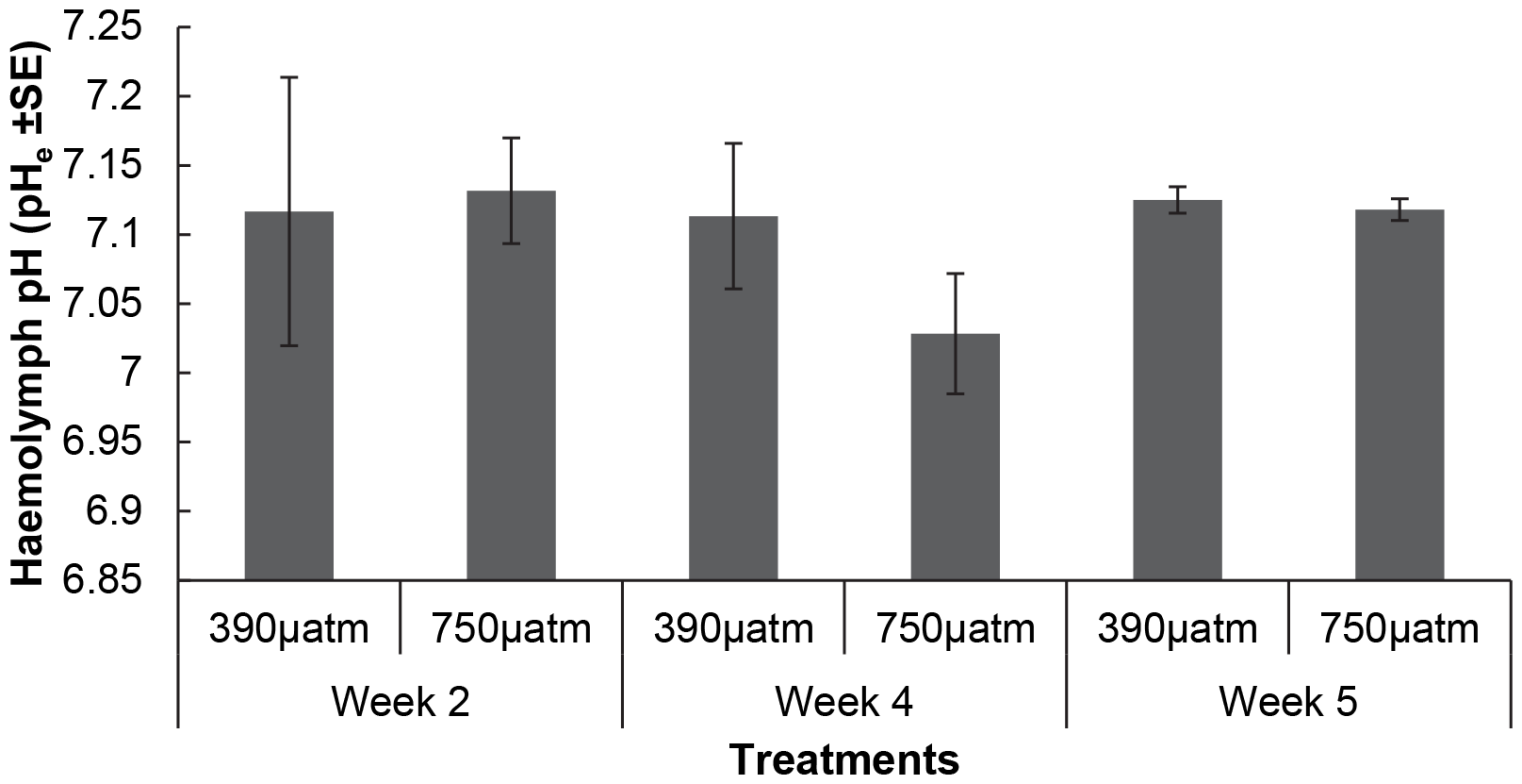
The mean haemolymph pH (pH_e_) of *M. asperrima* in response to ambient and elevated *p*CO_2_. Mean haemolymph pH after exposure to ambient and elevated *p*CO_2_ (390 µatm, 750 µatm), after exposure for 2, 4 and 5 weeks ±MSE, n = 3.

Overall there was a significant increase in the SMR of *M. asperrima* between two and four weeks of the experiment (Figure 4, Table 4), but there was no significant difference in SMR in response to elevated *p*CO_2_ levels at any sampling time (Table 4). There was, however, a significant effect of elevated *p*CO_2_ on the condition index. The combined mean of the condition index for all sampling times was greater in the ambient (390 µatm, mean ± SE = 16.2±0.5 n = 3) than the elevated *p*CO_2_ treatment (750 µatm, mean ± SE = 14.1±0.1 n = 3) (Figure 5, Table 4). Condition index was not, however, significantly different between treatments at week 4. There were also no significant differences in pH_e_ (Figure 6) and *p*_e_O_2_ (hPa) at elevated compared to ambient *p*CO_2_ at either sampling time (Table 4), although there were differences over time in *p*_e_O_2_ (Table 4).

**Table 4:** Summary of analysis of variance on *M. asperrima* adult physiological responses to elevated *p*CO_2_.

		Standard Metabolic Rate (SMR)			SMR (“Tank factor removed”)			pH_e_			*P_e_*O_2_			Condition Index			No. of byssal threads		
Source of variation	*df*	*MS*	*F*	*P*	*MS*	*F*	*P*	*MS*	*F*	*P*	*MS*	*F*	*P*	*MS*	*F*	*P*	*MS*	*F*	*P*
Time	2	0.9	4.8	*	0.92	3.72	*	0	0.9	ns	1147	15	**	2.3	0.68	ns			
*p*CO_2_	1	0.1	0.3	ns	0.08	1.34	ns	0	0.2	ns	13.53	0	ns	36	48	**	20.7	2.6	ns
Tank x *p*CO_2_	4	0.3	1.2	ns				0	1.6	ns	83.98	1	ns	0.8	0.23	ns	8.12	2.5	*
Time x *p*CO_2_	2	0.2	1.1	ns	0.22	0.88	ns	0	0.7	ns	180.2	2	ns	3.3	0.94	ns			
Time x Tank (*p*CO_2_)	8	0.2	1.7	ns				0	0.8	ns	78.94	1	ns	3.5	1.07	ns			

Summary of analysis of variance of the means of Standard Metabolic Rate (SMR), pH_e_, p_e_O_2_, condition index and number of byssal threads of *M. asperrima.* Significance level is indicated by asterisks, * *P*<0.05; ** *P*<0.01; *** *P*<0.001. SMR, pH_e_, *P_e_*O_2_, and condition index in response to two, four, and five weeks of elevated and ambient *p*CO_2_ (390, 750 µatm) were analysed using a 3 way ANOVA with “Time” and “*p*CO_2_” as fixed factors and “Tank” was nested in “*p*CO_2_”. SMR was also analysed with the “Tank” factor removed and “Time” and “*p*CO_2_” as fixed factors. Number of byssal threads in response to five weeks of elevated and ambient *p*CO_2_ (390, 750 µatm) was analysed using a 2 factor ANOVA with “Tank” nested in the fixed factor *“p*CO_2_”.

**Figure 7:**
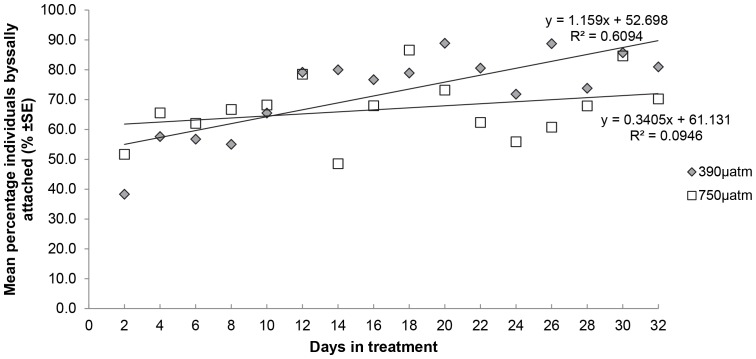
Mean Number of *M. asperrima* individuals byssally attached over experimental period. Mean byssal attachment is represented every 2 days over the 5 weeks of ambient (390 µatm) and elevated (750 µatm) treatments. Line represents linear regression with linear equation and R^2^ value n = 3.

While there was no significant difference in the amount of byssal threads per scallop between ambient and elevated *p*CO_2_ treatments after 5 weeks of exposure (Table 4), there were fewer byssal threads produced by scallops in the elevated (750 µatm, mean ± SE = 2.6±0.4 n = 3) compared to ambient (390 µatm, mean ± SE = 1.5±0.2 n = 3) treatments with some differences among tanks as shown by the “Tank” x “*p*CO_2_” interaction (Table 4). The percentage of scallops byssally attached over 5 weeks of exposure was dependent on the *p*CO_2_ treatment. The percentage of scallops byssally attached in the ambient treatment increased significantly over time, with a strong correlation (R^2^ = 0.6094, F_1, 14_ = 21.8, *P* = <0.001; Figure 7), but remained constant and not significant in the elevated (750 µatm) treatment (R^2^ = 0.0946, F_1, 14_ = 1.4, *P* = >0.05; Figure 7). After four days of exposure in the elevated treatment, 65% of scallops were attached, and after five weeks of exposure 66% of scallops were attached. In contrast, after four days in the ambient treatment 57% of scallops were attached and 81% of scallops were attached at the end of the five weeks of exposure (Figure 7).

## Discussion

### Fertilisation and Larvae

This study found that exposure to elevated *p*CO_2_ significantly affected the early life-history of *M. asperrima.* The fertilisation, development to trochophore stage and shell length of D-veliger larvae all decreased at elevated *p*CO_2_. The shell length of umbonate and pediveliger larvae and newly metamorphosed spat, however, were less sensitive to elevated CO_2_, with no significant reduction in shell length following acute exposure to elevated *p*CO_2_, and in some instances, positive effects of elevated *p*CO_2_ on shell growth. For many broadcast spawning marine organisms such as *M. asperrima*, fertilisation occurs in the water column and is directly affected by the environmental conditions [66]. Fertilisation is the first step in embryonic development, and is of particular importance to the reproductive success and population survival of marine free spawning invertebrates [67]. In this study, fertilisation of *M. asperrima* decreased with elevated *p*CO_2_. At the highest *p*CO_2_ level of 1000 µatm, the percentage fertilisation was reduced by 30%. Fertilisation of other marine organisms in response to elevated *p*CO_2_ has been shown to be quite robust [68], [69], although variable. Some studies on northern hemisphere bivalve populations have reported no effect of elevated *p*CO_2_ on the fertilisation of *C. gigas* [11], [70] and *M.s galloprovincialis* [28] at 2000 µatm *p*CO_2_. In contrast, when experiments were conducted on southern hemisphere populations, elevated *p*CO_2_ was found to reduce fertilisation in *C. gigas* and *S. glomerata* [12], [26]. The robustness of fertilisation has been questioned for other marine groups which are also broadcast spawners. For example, fertilisation was reduced during exposure to elevated *p*CO_2_ in the sea urchins, *Hemicentrotus pulcherrimus*, *Echinometra mathaei* [30], [71], and *Heliocidaris erythrogramma* [35]. However, in another study, when *H. erythrogramma* was collected from a similar location, Byrne [66] found that there was no difference in the percentage fertilisation under elevated *p*CO_2_ compared to ambient levels. Such contrasting results, from the same species sometimes from the same location, call into question the methodologies used to measure fertilisation [9], [26]. Byrne [72] has suggested a more consistent and integrated approach to facilitate accurate comparisons of fertilisation needs to be taken in future investigations.

Studies have questioned the effect of timing of exposure to elevated *p*CO_2_ on development, suggesting it is not until the trochophore stage and shell mineralisation (calcification) that the effects of elevated *p*CO_2_ will be observed [8], [11], [70]. Similar to this study, Parker et al. [12], [26] found there was a reduction in fertilisation of the oysters *S. glomerata* and *C. gigas* in response to elevated *p*CO_2_ just 2 h after sperm addition, which is well before the beginning of the trochophore stage. In a natural setting, mortality of marine invertebrates during their early development can often exceed 90% [73]. Exposure to stressors early in life can also elicit the “developmental domino effect” where the physiological performance of the earlier stages has severe implications on the success of the following ontogenetic stages [72]. A decrease in fertilisation and early development, as observed in this study, could have severe flow on effects to the number of settlers and affect settlement and recruitment [9].

The shell length of D-veliger larvae of *M. asperrima* decreased with increased *p*CO_2_ after four and six days of exposure. The effects of increased *p*CO_2_ on the size of D-veliger larvae has been observed for other bivalve species such as *M. edulis* [18] and *M. galloprovincialis* [28] the clam *Mercenaria mercenaria* [47] and gastropods such as the abalone *H. coccoriadata* [33]. Reductions in the size of larvae in response to elevated *p*CO_2_ has also been found in the sea urchins *H. pulcherrimus*, *E. mathaei* [30] *T. gratilla, Evechinus chloroticus*, and *Sterechinus neumayeri* [32], this is likely due to a decrease in larval calcification. Most studies report stunted larval development and decreased growth once larvae reach the calcifying stage [8], [11], [28]. Reductions in the calcification of adult marine organisms in response to elevated *p*CO_2_ are also widely documented [20], [74]–[78]. It has also been suggested by Stumpp et al. [79] that reductions in larval growth at elevated *p*CO_2_ can be attributed to a reduction of energy. Stumpp et al. [79] found decreased scope for growth in the larvae of the sea urchin *S. purpuratus* at elevated *p*CO_2_. In an attempt to combat the physiological effect of elevated the sea urchin larvae diverted energy away from somatic growth to homeostatic processes. The reduced energy available for growth meant that the sea urchin larvae at elevated *p*CO_2_ could not grow as fast as those under ambient *p*CO_2_ conditions.

The results of this study are consistent with others, finding a significant reduction in the percentage fertilisation [12], [26] and decreased size of D-veliger at elevated *p*CO_2_ [8], [11], [12], [26], [27], [36], [47]. The size of umbonate, pediveliger larvae and spat of *M. asperrima* were not, however, reduced at elevated *p*CO_2_, and at some stages growth may have been enhanced under elevated *p*CO_2_. This is in contrast to studies which have reported reduced shell length of umbonate, pediveliger and spat stages of the oysters *S. glomerata* and *C. gigas*, in response to elevated *p*CO_2_ [12], [26], [27]. Similar to *M. asperrima*, larvae of the bay scallop *Argopecten irradians* were only affected by elevated *p*CO_2_ (≈2000 µatm) on the first day of exposure [46] and similarly, White et al. [46] found that shell growth of larvae of *A. irradians* were reduced only up until day 7. Further, Talmage and Gobler [47] found larvae of *A. irradians* were only vulnerable in the very early life history stage and significantly less affected by elevated *p*CO_2_ compared to the clam *Mercenaria mercenaria*.

### Adults

It is known that as *p*CO_2_ increases, dissolved CO_2_ readily diffuses across the epithelial surfaces of marine molluscs and other marine organisms, where it equilibrates across the organism’s body spaces [23], [80] causing intra and extracellular acidification [23], [81]. If the organism cannot compensate, this acid-base balance disturbance can have detrimental consequences, including changes in energy metabolism [15]–[17], [37], [82], reductions in thermal tolerance and aerobic scope [83], compromises in immune responses [84], protein degradation [17], [85], decreases in somatic growth [15], [79], and in some extreme cases, increased mortality [86]–[89]. In an attempt to ameliorate these effects, organisms often try to increase their ion exchange to maintain acid-base balance. This requires energy, which can come at a cost to other physiological functions, or energy production (metabolic rate) is increased [15], [21]–[23], [43].

During the chronic five week exposure, adult *M. asperrima* showed no significant change in their standard metabolic rate (SMR), extracellular pH (pH_e_) and extracellular O_2_ (*p*_e_O_2_) in response to elevated *p*CO_2_. There were, however, significant effects on the condition and byssal attachment of adult *M. asperrima* over the duration of the exposure. The present study, like most other studies to date, measured the rate of respiration (SMR) of the whole organism. SMR of the whole organism provides an estimation of the overall sum of energy consuming processes, making it possible that small changes in a specific metabolic system may be overlooked. The absence of an alteration of the SMR (either an increase or decrease) of adult *M. asperrima* to elevated *p*CO_2_ in this study suggests that there was either (i) no change in metabolic functions in response to elevated *p*CO_2_ or (ii) a shift in the energy budget and metabolic functions, where a metabolic decrease in one tissue was compensated by a metabolic increase in another, giving no net change in SMR. The decline in condition index and byssal attachment suggests that latter (ii) had occurred. Schalkhausser et al. [48] found that the great scallop *Pecten maximus* also had no change in overall resting SMR when exposed to elevated *p*CO_2_, although it experienced a decrease in shell clapping power suggesting an energy trade off to maintain to maintain acid-base balance [48].

In this investigation, while there was a significant linear increase over time in the percentage of adult *M. asperrima* attached by byssal threads in the ambient treatment, there was no relationship between the percentage of adult *M. asperrima* attached in the elevated treatment. There was also a trend for the mean number of byssal threads secreted per animal to be greater in the ambient compared to the elevated treatment. Byssal attachment of scallops is known to be an energy demanding process, ranging between 4–14% of all somatic production [90], which is affected by environmental factors (mainly abiotic) and an indicator of physiological stress [91]–[96]. The relationship between stressful abiotic factors reducing byssal attachment such as increasing temperature and decreasing salinity, has been found for the Atlantic sea scallop *Placopecten magellanicus* [91], the great scallop *P. maximus* [92], *M. asperrima* [93], the queen scallop *Chlamys opercularis* [94], and the lions paw scallop *Nodipecten nodosus* [96]. Byssal attachment is also known to mirror the pattern of scallop growth and survival [93]. In this study, the decreased byssal attachment by *M. asperrima* suggests that elevated *p*CO_2_ is acting in a similar way to other abiotic factors such as salinity and temperature to cause a decrease in byssal attachment. The observed byssal attachment reduction is most likely the result of one or both of two mechanisms; a behavioural response and/or a metabolic response. Most bivalves close their valves in response to an environmental stressor such as salinity, scallops however, cannot seal their shell [97]. As a result species including *M. asperrima*, *A. irradians*, and *C. opercularis* attempt to escape via swimming. Byssal attachment obviously hinders the swimming response, so detachment allows the individual to swim away from the irritant, or to be washed away via currents [97]. The reduced percentage of scallops byssally attached may also be due to a metabolic trade-off to favour increased homeostatic functions.

The mode of life of *M. asperrima* may increase the capacity of this bivalve to cope with elevated *p*CO_2_ compared to more sessile species such as oysters and mussels. Water respiring aquatic organisms rely almost exclusively on ion exchange mechanisms to compensate for external CO_2_ induced disturbances in acid-base balance [21], [22], [43]. Melzner et al. [43] argues that organisms with a more active mode of life such as teleost fish, brachyurans, and cephalopods have a greater ability to regulate acid-base balance primarily due to their more advanced non-carbonate buffering system. It is presumed that these more advanced buffering mechanisms have developed to mitigate the acidifying effect of excess CO_2_ produced during exercise [43], [44]. During the rapid contractions of the adductor muscle associated with swimming, the heart rate of scallops can increase 2–3 fold, rising stroke volume and resulting in a total 5 fold increase in cardiac output [45]. This increased aerobic metabolism results in a spiking of CO_2_ in the haemolymph which must be dealt with. The scallop is the only bivalve which can facilitate locomotion on a relatively large scale, via this swimming response [97]. It may be possible, that scallops too have developed increased acid-base regulation to cope with elevated CO_2_ generated during their swimming response. An increased capacity to cope with elevated *p*CO_2_ within the circulatory system may explain why no change in SMR or pH_e_ was observed at rest in this investigation, yet such a response has been observed in more sessile bivalves [13], [16], [17], [48].

Overall, when adult *M. asperrima* were exposed to elevated *p*CO_2_ (750 µtm) there was no effect on SMR and pH_e_, but a significant reduction in the condition index and byssal attachment. The increased ion-exchange capacity of the scallops could possibly be more energy demanding resulting in a trade-off decreasing somatic growth [16], [17], [23], [79], as measured by a reduction in condition index. Decreased condition index under elevated *p*CO_2_ may also be due to reduction in protein turnover, resulting in lowered protein synthesis in some marine organisms [98]. Although sacrificing metabolic processes for the sake of homeostatic processes may be successful in the short term, it is likely that there are long term consequences for individuals and populations. Diverting energy away from and reducing somatic growth will potentially slow the reproductive development of scallops, which will in turn, affect reproductive output. If the response of *M. asperrima* is contrasted to studies on other Australian bivalves, Parker et al. [13] found *S. glomerata* significantly increased SMR, significantly decreased pH_e_ with no effect on condition index when exposed to elevated *p*CO_2_ of 750 µatm.

Previous investigations have confirmed the ability of intertidal organisms to adapt and respond to elevated *p*CO_2_ with increased resilience if they are previously exposed to a low pH or fluctuating environment [13], [31], [32], [37]. These results prompted the hypothesis that those organisms from stable pH environments such as the subtidal, would be more vulnerable to elevated *p*CO_2_. This study does not support this hypothesis. Although *M. asperrima* were negatively affected across many life history stages by elevated CO_2_, *M. asperrima* (and most other scallop species previously investigated [46], [47], [48]) were not affected to the extent that has been reported for intertidal bivalves such as *M. edulis*, *M. galloprovincialis* [18], [28] *S. glomerata* and *C. gigas* [12], [13], [16], [26]. Benthic respiration as suggested by Miller et al. [36] or similar environmental processes may allow *M. asperrima* and other scallops to develop a capacity to cope with an elevated *p*CO_2_ environment, although measurement of benthic respiration and CO_2_ flux monitoring is required to confirm this.

This study has found that fertilisation and early larval development of the economically and ecologically significant *M. asperrima* decreased with elevated *p*CO_2_. Later larval stages were not as affected by elevated *p*CO_2_ as early larval stages and some stages may have experienced enhanced growth when exposed to elevated *p*CO_2_. It appears that *M. asperrima* larvae were less sensitive to elevated *p*CO_2_ than the larval stages of the estuarine oysters *S. glomerata* and *C. gigas* [12], [13], [16], [26]. After a five week exposure to elevated *p*CO_2_ adult *M. asperrima* were also in poorer condition and had decreased byssal attachment. The results of this study suggest that species responses to elevated *p*CO_2_ are complex, and when attempting to predict and explain their response, the physiochemical characteristics of the environment, mode of life and responses across life history stages need to be considered. Even with the more moderate set of responses measured in this study the effects of ocean acidification are still likely to reduce the abundance of *M. asperrima* and other scallop species in the world’s oceans.
